# Supplementary material for: Strategic Doping for Precise Structural Control and Intense Photocurrents Under Visible Light in Ba2M0.4Bi1.6O6 (M = La, Ce, Pr, Pb, Y) Double Perovskites
Source: Nanomaterials (Basel). 2025 Jul 4;15(13):1039. doi: 10.3390/nano15131039 (PMC12251094; doi:10.3390/nano15131039)
Supplement: Supplementary file 1 [file nanomaterials-15-01039-s001.zip › nanomaterials-3646289-supplementary.pdf]

Supplementary Materials

Strategic Doping for Precise Structural Control and Intense Photocurrents Under Visible Light in $Ba_2M_{0.4}Bi_{1.6}O_6$ (M = La, Ce, Pr, Pb, Y) Double Perovskites

Tirong Guo, Wen Tian Fu and Huub J. M. de Groot *

Leiden Institute of Chemistry, Leiden University, Einsteinweg 55, 2300 RA Leiden, The Netherlands; t.guo@lic.leidenuniv.nl (T.G.); w.fu@chem.leidenuniv.nl (W.T.F.)

* Correspondence: groot_h@lic.leidenuniv.nl

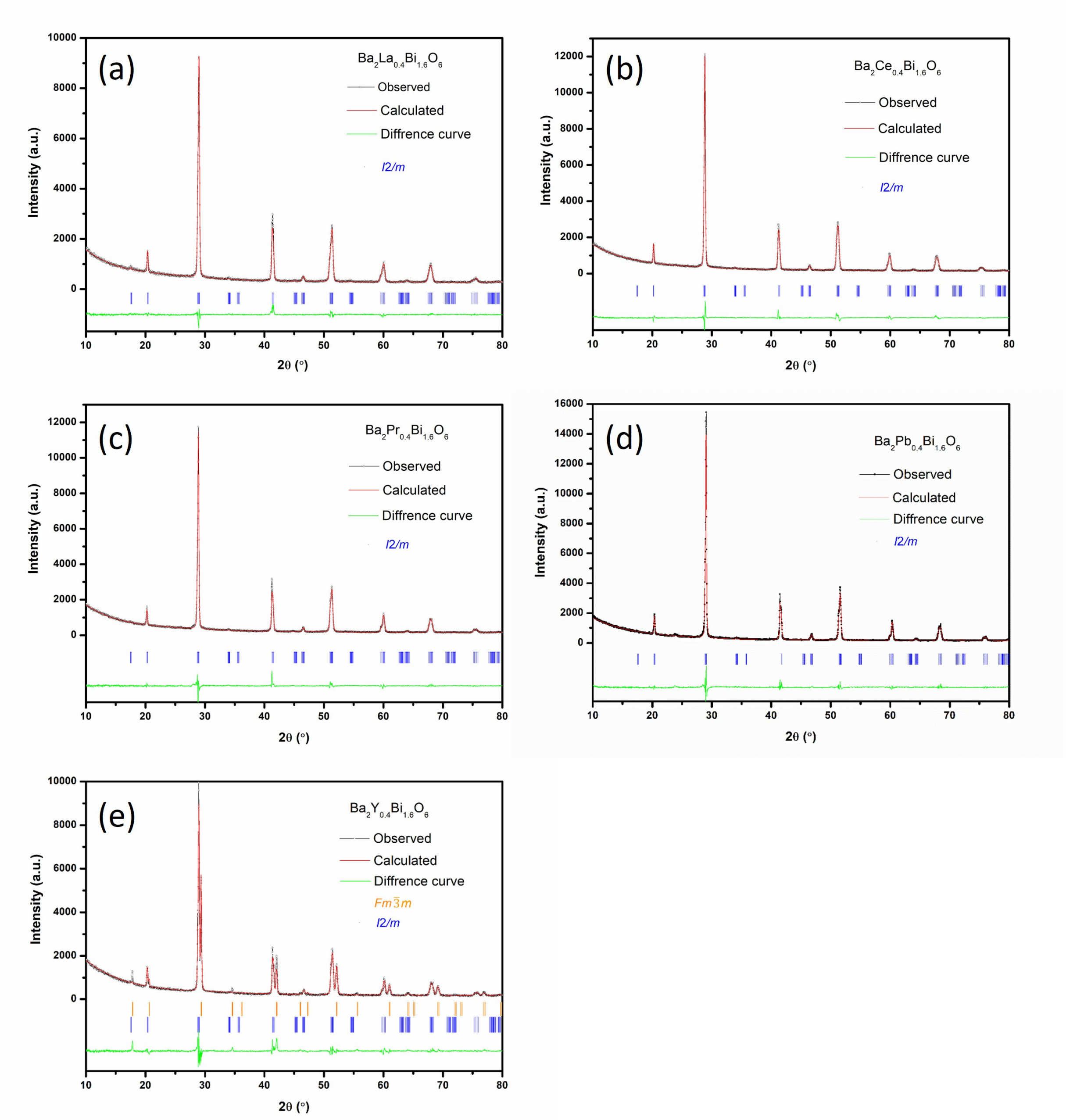

 https://doi.org/10.3390/nano15131039

Figure S1. Rietveld refinement profiles for $Ba_2M_{0.4}Bi_{1.6}O_6$(M=La, Ce, Pr, Pb, Y) powder XRD patterns based on the single-phase monoclinic space group *I*2/*m* in panel (a) (b) (c) and (d); and double phases including the cubic space group $Fm\bar{3}m$ for $Ba_2Y_{0.4}Bi_{1.6}O_6$ and the monoclinic space group *I*2/*m* for $Ba_2Bi_2O_6$ in panel (e).

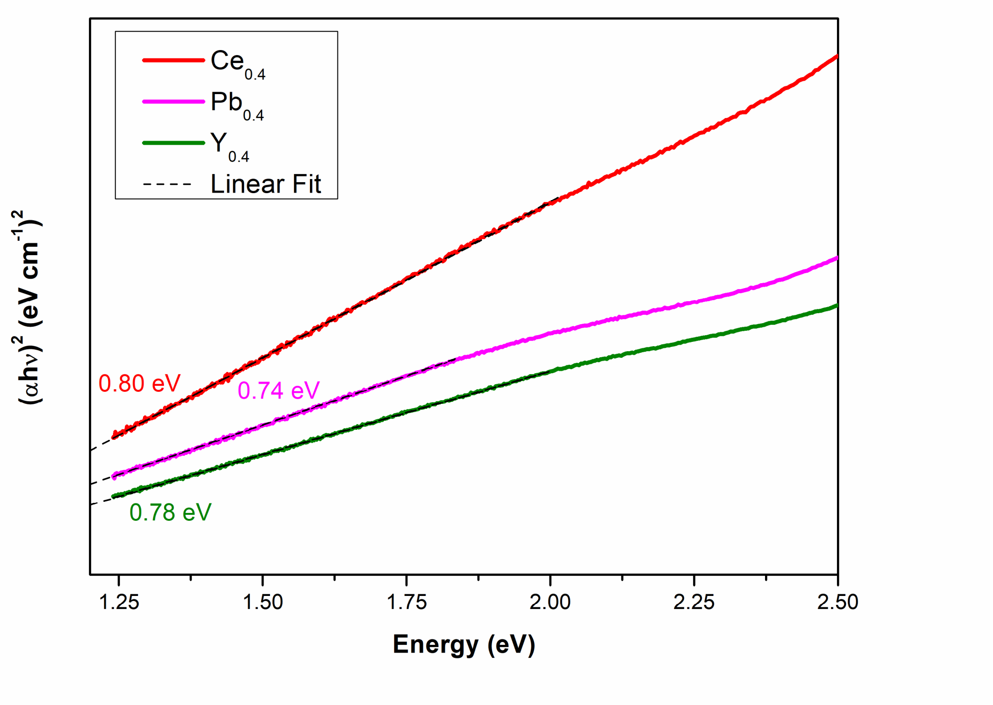

Figure S2. The Tauc plot linear fitting results for $Ba_2M_{0.4}Bi_{1.6}O_6$(M=Ce, Pb, Y) correlated to the long light absorption tails.

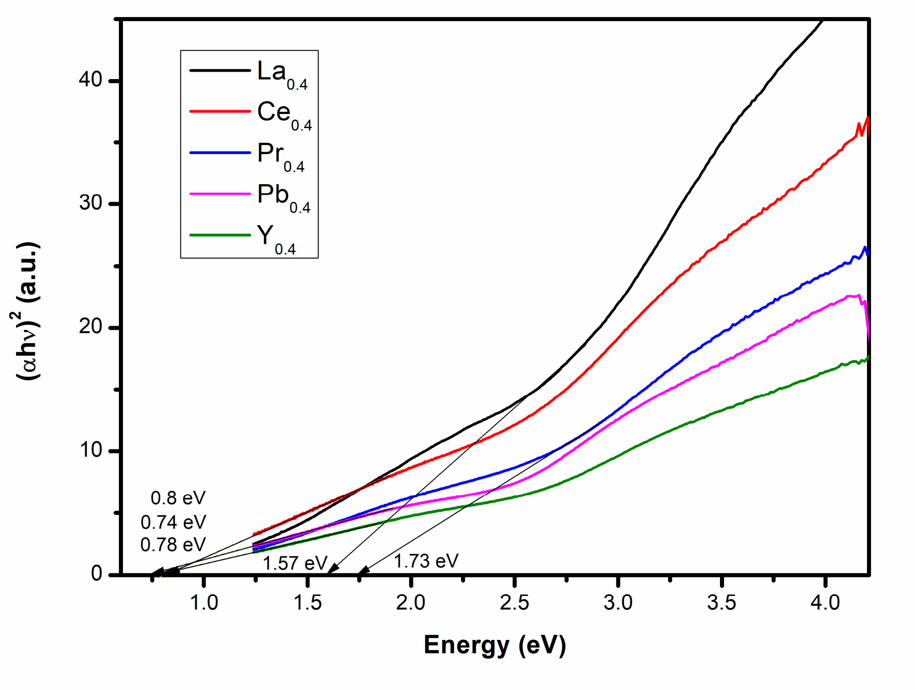

Figure S3. The overview of the Tauc plot with linear fitting in the first linear region correlating to the onset of optical absorption in double perovskites $Ba_2M_{0.4}Bi_{1.6}O_6$(M=La, Ce, Pr, Pb, Y).

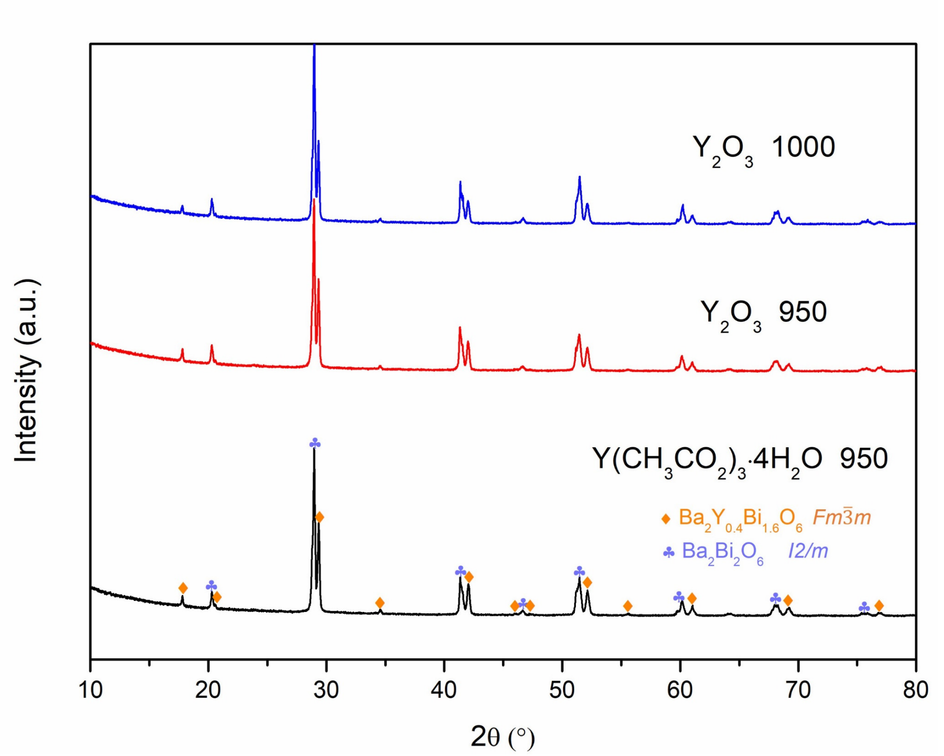

Figure S4. XRD results for $Ba_2M_{0.4}Bi_{1.6}O_6$(M=Y) powders prepared from the starting reagent $Y(CH_3CO_2)_3{\cdot}4H_2O$ and calcined at 950 °C (in black trace); and prepared from the starting reagent Y_2O_3 and calcined at 950 °C (in red trace) and 1000 °C (in blue trace). All powders show the mixed dual double perovskite phases composition in space group $Fm\bar{3}m$ and *I*2/*m*.

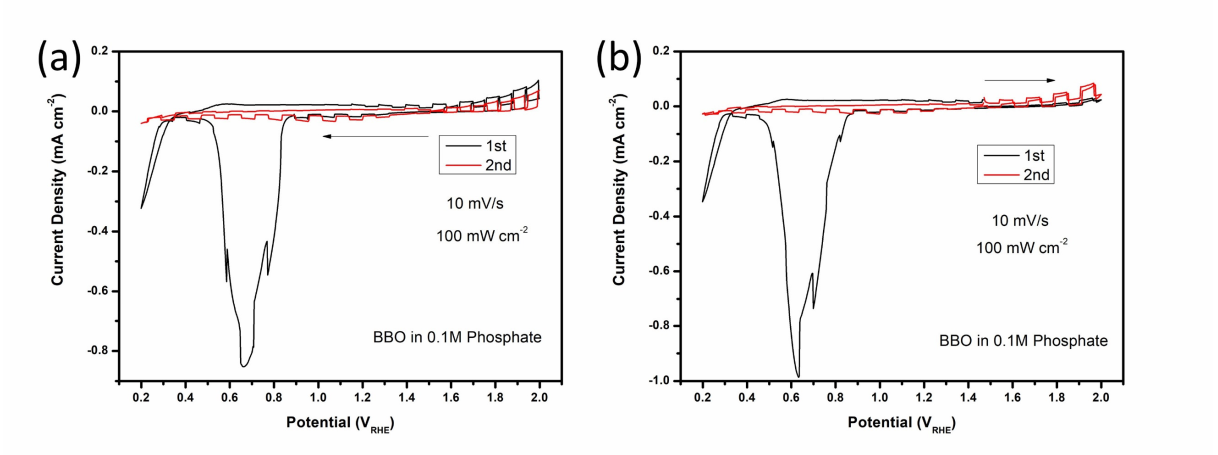

Figure S5. CV patterns of the parent BBO thin film photoelectrodes recorded starting from the OCP potential (a) with the cathodic scan and (b) with the anodic scan under periodic 1 sun's illumination.

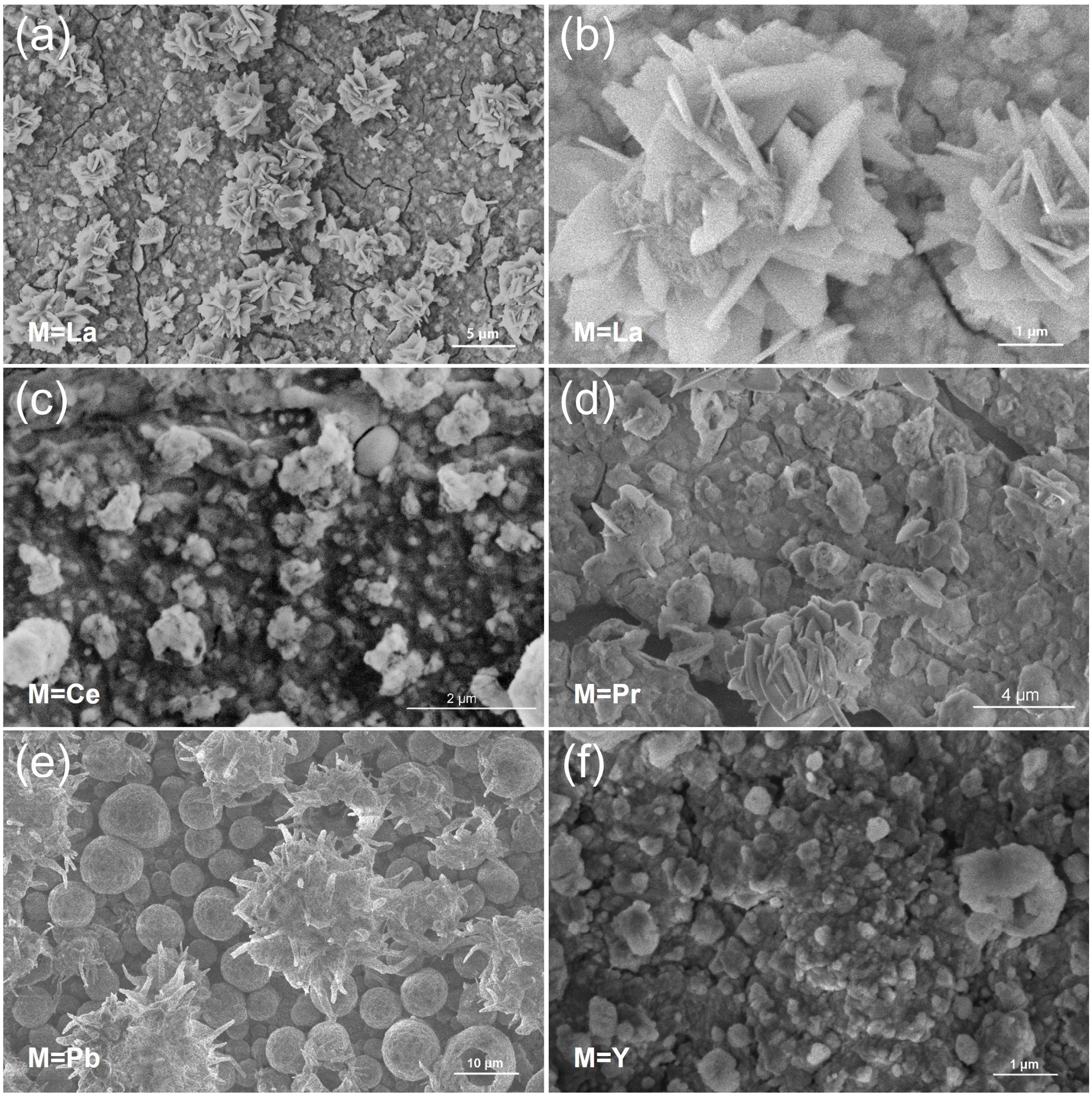

Figure S6. SEM surface morphologies of the dip-coated $Ba_2M_{0.4}Bi_{1.6}O_6$ photoelectrodes for M= (a) (b)La, (c) Ce, (d) Pr, (e) Pb and (f) Y after illumination chopped LSV measurements.

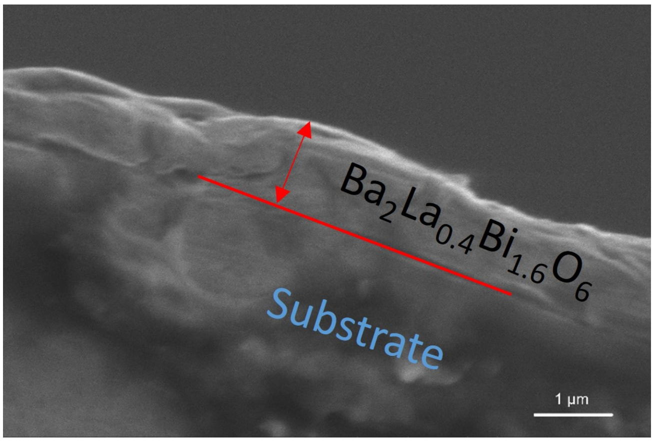

Figure S7. The cross-sectional image of photoelectrode $Ba_2M_{0.4}Bi_{1.6}O_6$ for M=La prepared by 4-times spin coating.

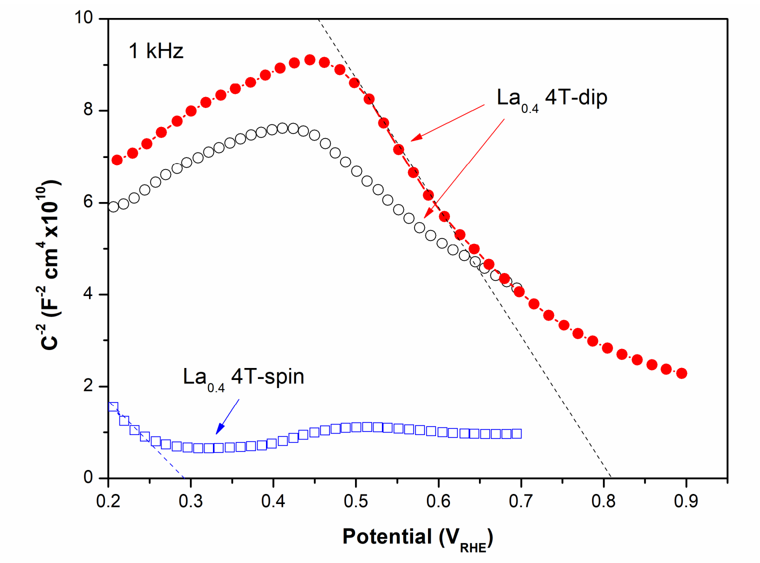

Figure S8. Mott-Schottky plot of the $Ba_2La_{0.4}Bi_{1.6}O_6$ photoelectrodes prepared by the dip-coating method (red dots and black circles) and the spin-coating method (blue squares) in the dark with the neutral 0.1 M sodium phosphate buffer electrolyte.
